# Expression patterns, molecular markers and genetic diversity of insect-susceptible and resistant *Barbarea* genotypes by comparative transcriptome analysis

**DOI:** 10.1186/s12864-015-1609-y

**Published:** 2015-07-01

**Authors:** Xiaohui Zhang, Tongjin Liu, Xiaochun Wei, Yang Qiu, Jiangping Song, Haiping Wang, Di Shen, Niels Agerbirk, Xixiang Li

**Affiliations:** Institute of Vegetables and Flowers, Chinese Academy of Agricultural Sciences; Key Laboratory of Biology and Genetic Improvement of Horticultural Crops, Ministry of Agriculture, Beijing, 100081 China; Institute of Horticulture, Henan Academy of Agricultural Sciences, Zhengzhou, 450002 China; Department of Plant and Environmental Sciences, University of Copenhagen, Thorvaldsensvej 40, DK-1871 Frederiksberg C, Denmark

**Keywords:** *Barbarea vulgaris*, Transcriptome, Diamondback moth, Expression pattern, Molecular marker, Genetic diversity, Saponin biosynthesis

## Abstract

**Background:**

*Barbarea vulgaris* contains two genotypes: the glabrous type (G-type), which confers resistance to the diamondback moth (DBM) and other insect pests, and the pubescent type (P-type), which is susceptible to the DBM. Herein, the transcriptomes of P-type *B. vulgaris* before and after DBM infestation were subjected to Illumina (Solexa) pyrosequencing and comparative analysis.

**Results:**

5.0 gigabase pairs of clean nucleotides were generated. Non-redundant unigenes (33,721) were assembled and 94.1 % of them were annotated. Compared with our previous G-type transcriptome, the expression patterns of many insect responsive genes, including those related to secondary metabolism, phytohormones and transcription factors, which were significantly induced by DBM in G-type plants, were less sensitive to DBM infestation in P-type plants. The genes of the triterpenoid saponin pathway were identified in both G- and P-type plants. The upstream genes of the pathway showed similar expression patterns between the two genotypes. However, gene expression for two downstream enzymes, the glucosyl transferase (*UGT73C11*) and an oxidosqualene cyclase (*OSC*), were significantly upregulated in the P-type compared with the G-type plant. The homologous genes from P- and G-type plants were detected by BLAST unigenes with a cutoff level E-value < e^−10^. 12,980 gene families containing 26,793 P-type and 36,944 G-type unigenes were shared by the two types of *B. vulgaris.* 38,397 single nucleotide polymorphisms (SNPs) were found in 9,452 orthologous genes between the P- and G-type plants. We also detected 5,105 simple sequence repeats (SSRs) in the *B. vulgaris* transcriptome, comprising mono-nucleotide-repeats (2,477; 48.5 %) and triple-nucleotide-repeats (1,590; 31.1 %). Of these, 1,657 SSRs displayed polymorphisms between the P- and G-type. Consequently, 913 SSR primer pairs were designed with a resolution of more than two nucleotides. We randomly chose 30 SSRs to detect the genetic diversity of 32 *Barbarea* germplasms. The distance tree showed that these accessions were clearly divided into groups, with the G-type grouping with available Western and Central European *B. vulgaris* accessions in contrast to the P-type accession, *B. stricta* and *B. verna*.

**Conclusions:**

These data represent useful information for pest-resistance gene mining and for the investigation of the molecular basis of plant-pest interactions.

**Electronic supplementary material:**

The online version of this article (doi:10.1186/s12864-015-1609-y) contains supplementary material, which is available to authorized users.

## Background

*Barbarea* is a genus comprising 29 species of flowering plants from the tribe Cardamineae in the family Brassicaceae [1]. *B. vulgaris*, *B. intermedia*, *B. stricta*, and *B. verna* are four widely distributed biennial species that are commonly used as model plants in research [2, 3]. *B. vulgaris* contains two distinct genotypes: the G-type has glabrous leaves and is resistant to infestation by the diamondback moth (DBM, *Plutella xylostella*) and a flea beetle (*Phyllotreta nemorum*); the P-type has pubescent leaves and is susceptible to DBM and flea beetles [4–6]. The two types also differ in their glucosinolate composition, flavonoid composition, saponin content and disease susceptibility [4–11]. Recently, significant genetic diversity and reproductive incompatibility have been reported between the two plant types [12]. The ability to induce oviposition and kill larvae of the DBM, a serious pest of cruciferous crops, makes the G-type *B. vulgaris* a useful trap crop for pest management [13–16]. The P- and G-types of *B. vulgaris* and the DBM represent an ideal model to study evolution in plant-insect ecology [4–21].

The insect resistance ability of G-type *B. vulgaris* relies on the four triterpenoid saponins, including 3-*O*-cellobiosyl-oleanoic acid, 3-*O*-cellobiosyl-hederagenin, 3-*O*-cellobiosyl-gypsogenin and 3-*O*-cellobiosyl-4-epihederagenin [5, 18–20]. To the best of our knowledge, these metabolites are not found in P-type *B. vulgaris* or non-*Barbarea* cruciferous plants. The saponin biosyntheses have developed from the triterpenoid pathway, which exists commonly in plants. The triterpenoid backbone, 2,3-oxidosqualene, is first cyclized to form a core structure by oxidosqualene cyclases (OSCs), and then decorated by cytochrome P450s (P450s) and further covalently linked to sugars by glycosyltransferases (UGTs) [21]. The different decorations and diverse sugar residues of saponins produce distinct biological activities, such as anti-pathogenic, insecticidal, anti-tumorigenic and immunomodulatory effects [22–24]. In G-type *B. vulgaris*, the 2,3-oxidosqualene is cyclized into β-amyrin by a specific OSC, and then the C23, C24 and C28 residues are modified by P450s to form the four sapogenins, all of which are subsequently linked to a cellobiose at the C3 position with a carbon–oxygen β-glycosidic bond [5, 19, 20], thus conferring insect deterrence or insecticidal activity [25]. The UGTs catalyzing the first glycosylation have been cloned from both G- and P-type *B. vulgaris* [25]*.* However, the P450s responsible for the C23, C24 and C28 decoration and the UGTs for the second glycosylation remain unidentified.

Much research effort has been applied to identify the anti-insect genes in *B. vulgaris*, including quantitative trait locus (QTL) mapping and cDNA scanning. A P × G-type derived F2 population has been used for QTL analysis; the insect resistance and the four kinds of triterpenoid saponins co-segregated and were mapped on two genomic regions spanning 7–30 cM [26]. Higher-density markers are needed for fine mapping and finally cloning of the resistance genes. By activity scanning of a cDNA expression library, *UGT73C11* encoding the enzyme catalyzing the first monoglucosylation of the saponins was identified. The authors also detected two homologous genes from both P- and G-type *B. vulgaris* with similar functions [25]. Another efficient technology to identify saponin pathway genes is transcription profiling. Using cDNA-amplified fragment length polymorphism-based transcript profiling of jasmonate-treated plant tissues, a regulator gene *MKB1* has been screened out in *Medicago truncatula* and its control mechanism to manage the biosynthesis of triterpene saponins was further revealed [27]. Using a similar method, a synthesis enzyme of the saponin biosynthesis pathway, CYP716Y1, has been identified in *Bupleurum falcatum*, and combining this with several other genes the synthesis of monoglycosylated saponins was reconstituted in yeast [28]. Benefitting from the rapid development of next-generation DNA sequencing technology, pyrosequencing has become a labor-efficient and cost-saving transcript profiling method.

Our previous study revealed the transcriptome profile of G-type *B. vulgaris* under a series of DBM herbivory treatments [29]. The DBM-responsive genes, including those related to secondary metabolism, transcription factors and signaling transduction, were monitored. In the present study, we report the transcriptomes of P-type *B. vulgaris* before and under DBM infestation, with the aim of determining the differential expression patterns between the two plant genotypes in response to the DBM. The saponin biosynthesis pathways of the two genotypes were compared. A rich set of single nucleotide polymorphism (SNP) and simple sequence repeat (SSR) markers were identified, which will accelerate the map-based cloning of the resistance genes. Additionally, the quality of the SSRs was tested experimentally and the genetic diversity of the *Barbarea* plants analyzed.

## Results

### Generation and annotation of the transcriptomes of P-type *Barbarea vulgaris*

The transcriptomes of G-type *B. vulgaris* under diamondback moth feeding and non-feeding conditions were pyrosequenced in our previous study [29]. To perform a comparative analysis of the transcriptomes between susceptible and resistant genotypes of *Barbarea vulgaris*, the transcriptomes of P-type seedlings under DBM feeding and non-feeding conditions were pyrosequenced in the present study. A total of 13,684,884 and 11,415,972 clean paired-end reads containing 2.764 and 2.238 gigabase pairs of clean nucleotides were generated from insect infested and control P-type *B. vulgaris*, respectively. These data were assembled into a set of 33,721 non-redundant unigenes, with a mean length of 896 nt and an N50 length of 1,440 nt, which were comparable with the G-type transcriptome assembly (Table 1). The length distribution of the unigenes are listed in Additional file 1: Table S1.

**Table 1:** Summary of the sequencing and assembly of the G- and P-type B. vulgaris transcriptomes

Genotype	G-type		P-type	
Treat	Control	Infested	Control	Infested
Clean reads	26,590,648	79,502,334	11,415,972	13,684,884
Clean nucleotides (Mb)	2,393	7,155	2,238	2,764
Unigene	39,531		33,721	
Mean length (nt)	815		896	
N50 length (nt)	1,175		1,440	
All_Annotated/n (%)	37,780 (95.6 %)		31,715 (94.1 %)	
nt_Annotation/n (%)	36,997 (93.6 %)		29,339 (87.0 %)	
nr_Annotation/n (%)	36,133 (91.4 %)		28,819 (85.5 %)	
Swissprot/n (%)	22,588 (57.1 %)		23,045 (68.3 %)	
GO/n (%)	14,339 (36.4 %)		24,113 (71.5 %)	
COG/n (%)	13,118 (33.2 %)		10,646 (31.6 %)	
KEGG/n (%)	19,620 (49.6 %)		8,167 (24.2 %)	

*GO*, gene ontology; *COG*, clusters of orthologous groups; *KEGG*, Kyoto encyclopedia of gene and genomes

**Fig. 1:**
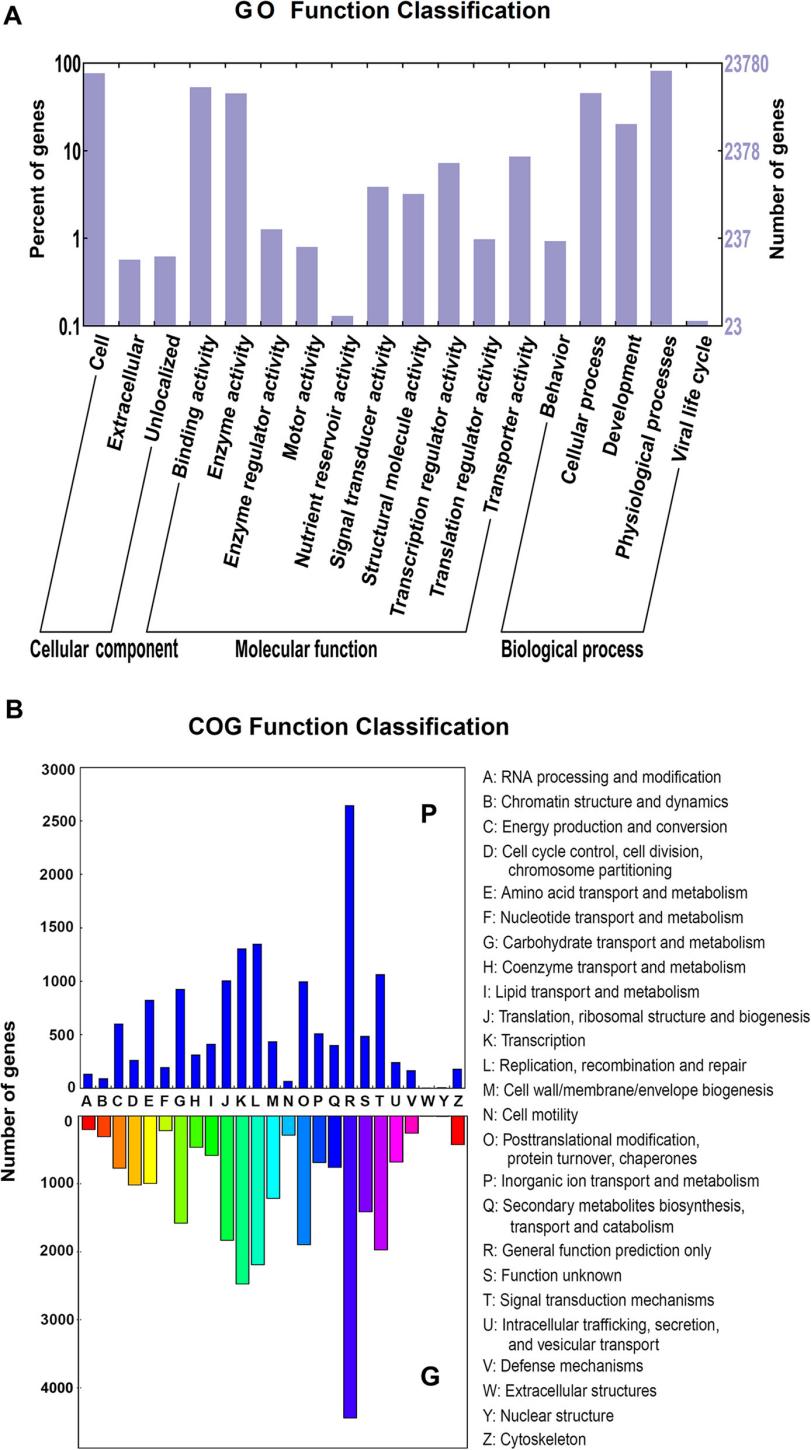
Function classification of unigenes. **a**, The gene ontology (GO) classification of P-type *B. vulgaris* transcripts; **b**, The comparison of clusters of orthologous groups of proteins (COGs) classification between P- and G-type transcriptomes

Subsequently, we screened the unigene sequences against the NCBI non-redundant (Nr), SwissProt, Gene Ontology (GO), Clusters of Orthologous Groups of proteins (COGs), and Kyoto Encyclopedia of Genes and Genomes (KEGG) pathway protein databases using BLASTX (*e*-value < 0.00001). Protein function was predicted from the annotations of the most similar proteins in those databases. As shown in Table 1, 31,715 (94.1 %) unigenes were annotated by at least one of these databases. Detailed information on the Nt, Nr, SwissProt, GO, COGs and KEGG annotations is shown in Tables S2–S7, respectively. The gene functional classification by GO analysis showed that the largest GO terms were “cell”, “binding activity” and “physiological processes” from the “cellular component”, “molecular function” and “biological process” ontologies, respectively. The most abundant COGs terms were “general function prediction only”, “replication, recombination and repair” and “transcription”. The distributions of the functional categories were similar to that of G-type plants (Fig. 1).

### Comparison of pest-induced transcriptome patterns between susceptible and resistant *B. vulgaris*

**Fig. 2:**
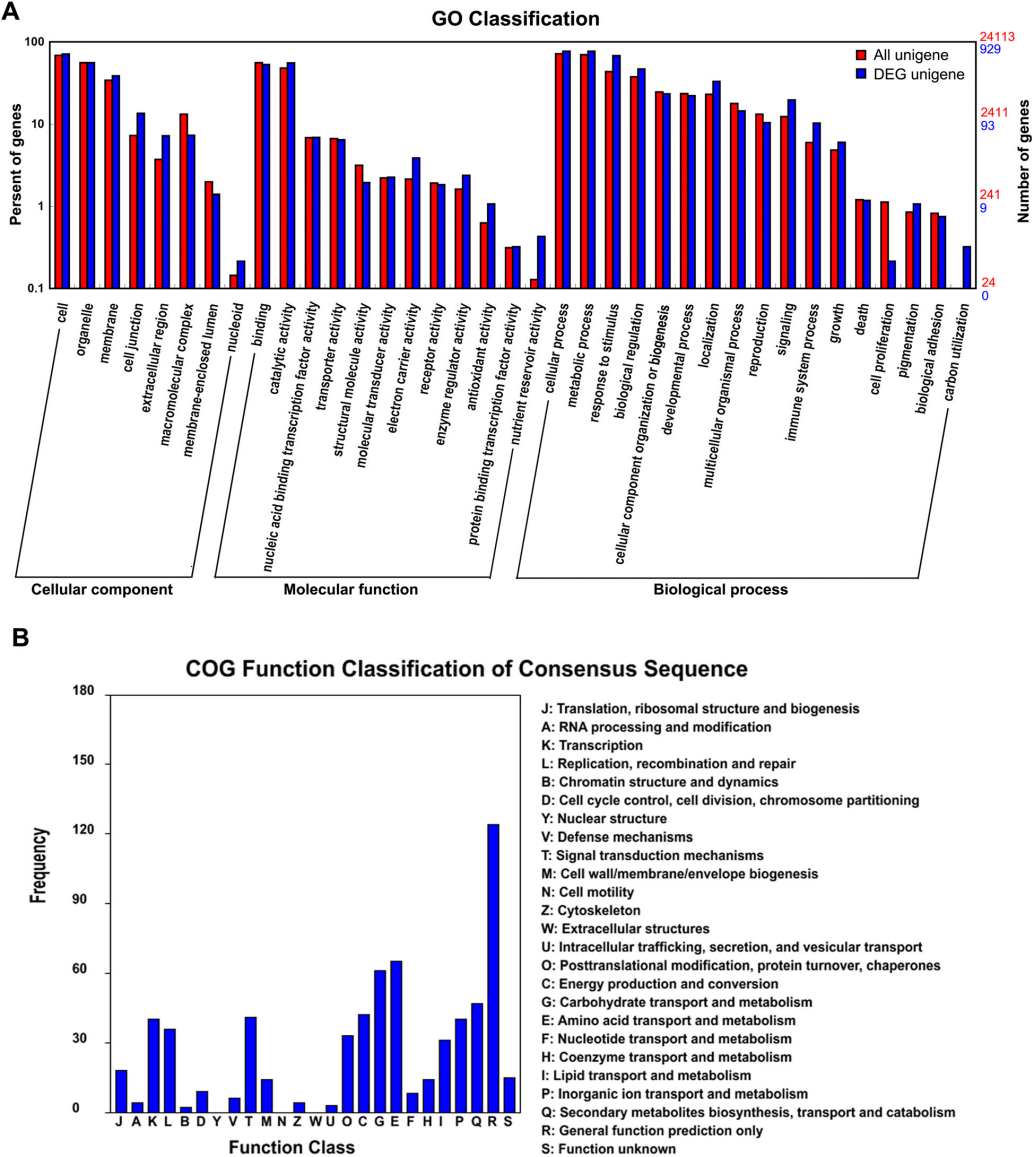
The functional classification of differentially expressed unigenes in P-type *B. vulgaris* infested with DBM. **a**, gene ontology (GO); **b**, clusters of orthologous groups of proteins (COGs)

Of the unigenes, 1,029 were differentially expressed in P-type *B. vulgaris*, including 530 up- and 499 downregulated, by DBM infestation (Additional file 1: Table S8; Additional file 2: Figure S1). This is far fewer than the number of genes affected by DBM in G-type plants, which accounted for 2,102–4,685 up- and 1,254–5,188 downregulated genes at a series of experimental time points [29]. The GO classification of the differentially expressed P-type unigenes indicated that the “cell junction” and “extracellular region”, “electron carier activity” and “enzyme regulator activaty”, “response to stimulus” and “localization” were the over-represented terms from the “cellular component”, “molecular function” and “biological process” ontologies, respectively. The most abundant COG class was “general function prediction only”, followed by “amino acid transport and metabolism” and “carbohydrate transport and metabolism” (Fig. 2). KEGG pathway analysis indicated that the over-represented pathways of the DBM-infected P-type transcriptome were “Nitrogen metabolism”, “Phenylpropanoid biosynthesis”, “Photosynthesis–antenna proteins” and “Flavonoid biosynthesis” (Table 2 and Additional file 1: Table S9). As shown in Additional file 2: Figure S2, the photosynthesis pathway genes were generally repressed by DBM infestation, indicating that the pest not only consumed the existing assimilates, but also disrupted the photosynthesis process. The phenylpropanoid biosynthesis and flavonoid biosynthesis pathways were upregulated extensively (Additional file 2: Figures S3 and S4), similar to the results found in G-type *B. vulgaris* and Arabidopsis [29, 30], indicating that these kinds of secondary metabolites [11] were common response compounds to DBM infestation in plants. Generally, in susceptible plants, the main DBM-affected genes are those engaged in nutrient, amino acid, carbohydrate transport and metabolism, and photosynthetic processes. However, genes related to certain secondary metabolism pathways such as glucosinolate biosynthesis, as well as phytohormones and transcription factors, which were dramatically induced by DBM in G-type *B. vulgaris*, showed less significant induction in P-type plants.

**Table 2:** The Kyoto encyclopedia of gene and genomes (KEGG) pathways affected by DBM in P-type B. vulgaris

Kegg_pathway	ko_id	DifferentFr N out of 758	GenomeFr N out of 14269	P-value	Corrected P-value
Nitrogen metabolism	ko00910	17 (2.24 %)	80 (0.56 %)	7.75E-7	1.56E-4
Phenylpropanoid biosynthesis	ko00940	14 (1.85 %)	86 (0.60 %)	1.62E-4	0.0326
Photosynthesis - antenna proteins	ko00196	11 (1.45 %)	57 (0.40 %)	1.73 E-4	0.0348
Flavonoid biosynthesis	ko00941	7 (0.92 %)	25 (0.18 %)	2.40 E-4	0.0483
alpha-Linolenic acid metabolism	ko00592	8 (1.06 %)	38 (0.27 %)	7.21 E-4	0.145
Phenylalanine metabolism	ko00360	11 (1.45 %)	78 (0.55 %)	2.65E-3	0.533
Mineral absorption	ko04978	4 (0.53 %)	12 (0.08 %)	2.78 E-3	0.558
Sesquiterpenoid biosynthesis	ko00909	2 (0.26 %)	2 (0.014 %)	2.82 E-3	0.567
Stilbenoid, diarylheptanoid and gingerol biosynthesis	ko00945	7 (0.92 %)	43 (0.30 %)	7.02 E-3	1
Alanine, aspartate and glutamate metabolism	ko00250	9 (1.19 %)	65(0.46 %)	7.11 E-3	1
Circadian rhythm - plant	ko04712	6 (0.79 %)	33 (0.23 %)	7.13 E-3	1
Aminobenzoate degradation	ko00627	7 (0.92 %)	47 (0.33 %)	0.0114	1
Valine, leucine and isoleucine biosynthesis	ko00290	10 (1.32 %)	83 (0.58 %)	0.0123	1
Glycan binding proteins	ko04091	4 (0.53 %)	18 (0.13 %)	0.0133	1
D-Glutamine and D-glutamate metabolism	ko00471	2 (0.26 %)	4 (0.028 %)	0.0157	1
Glucosinolate biosynthesis	ko00966	3 (0.39 %)	12 (0.084 %)	0.0229	1
Arginine and proline metabolism	ko00330	11(1.45 %)	105( 0.74 %)	0.0235	1
Sulfur metabolism	ko00920	6 (0.79 %)	43 (0.30 %)	0.0251	1
Photosynthesis proteins	ko00194	16 (2.11 %)	180 (1.26 %)	0.0303	1
beta-Alanine metabolism	ko00410	6 (0.79 %)	48 (0.34 %)	0.0405	1
Tryptophan metabolism	ko00380	8 (1.06 %)	75 (0.53 %)	0.0450	1
Zeatin biosynthesis	ko00908	3 (0.40 %)	16 (0.11 %)	0.0498	1
Protein processing in endoplasmic reticulum	ko04141	18 (2.37 %)	223 (1.56 %)	0.0509	1

DifferentFr, differentially expressed frequency, indicating the number and percentage of differentially expressed genes in each cluster; GenomeFr, genome frequency, indicating the number and percentage of the transcriptome distributed in each cluster.

### The saponin pathway in susceptible and resistant *B. vulgaris*

**Fig. 3:**
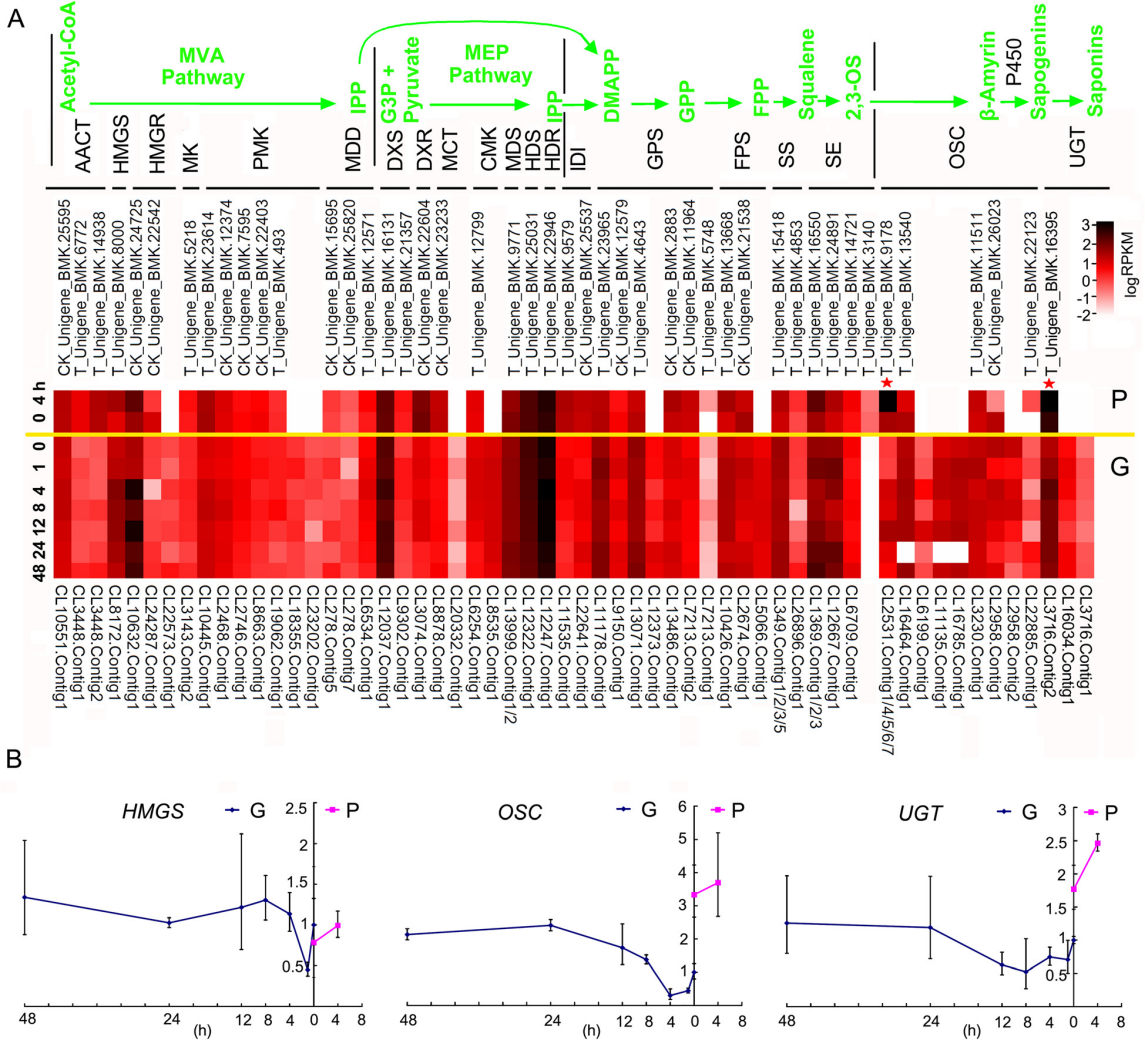
Comparative transcription profiles of the putative genes in the triterpene saponin synthetic pathway in P- and G-type *B. vulgaris*. **a**, Heatmaps representing the expression levels of different genes/families determined by RNA-seq. The color bar is shown on the top right. Data represent log (RPKM) of each treatment. The metabolites of the pathway are shown in bright green on top and the enzymes for each metabolic step are shown below in black using the following abbreviations: MVA, mevalonic acid; MEP, 2-*C*-methyl-D-erythritol 4-phosphate; IPP, isopentenyl diphosphate; G3P, glyceraldehyde 3-phosphate; DMAPP, dimethylallyl diphosphate; GPP, geranyl diphosphate; FPP, farnesyl diphosphate; 2,3-OS, 2,3-oxidosqualene; AACT, acetyl-CoA acyltransferase; HMGS, 3-hydroxy-3-methylglutaryl CoA synthase; HMGR, 3-hydroxy-3-methylglutaryl CoA reductase; MK, mevalonic acid kinase; PMK, phosphomevalonate kinase; MDD, mevalonic acid diphosphate decarboxylase; DXS, deoxy-D-xylulose 5-phosphate synthase; DXR, deoxy-D-xylulose 5-phosphate reductase; MCT, 2-*C*-methyl-D-erythritol 4-phosphate cytidylyltransferase; CMK, 4-diphosphocytidyl-2-*C*-methyl-D-erythritol kinase; MDS, 2-*C*-methyl-D-erythritol 2,4-cyclodiphosphate synthase; HDS, (*E*)-4-Hydroxy-3-methyl-but-2-enyl pyrophosphate (HMB-PP) synthase; HDR, HMB-PP reductase; IDI, IPI isomerase; GPS, geranylgeranyl pyrophosphate synthase; FPS, farnesyl pyrophosphate synthase; SS, squalene synthase; SE, squalene epoxidase; OSC, oxidosqualene cyclases; P450, cytochrome P-450; UGT, UDP-dependent glycosyl transferases. Two stars indicate the two significantly induced genes in P-type plants. **b**, Q-PCR analysis of three selected genes of the pathway. The ordinates show the relative expression levels; the abscissas show the time points. Error bars indicate the SD of three biological replicates

The genes of the triterpenoid saponin pathway (except *P450s*) were identified in both G- and P-type *B. vulgaris* transcriptomes, based on gene annotation. To limit the omissions, the saponin pathway genes were BLAST searched against the transcriptome databases from both G- and P-type plants. Seventy-one G-type and 44 P-type unigenes representing 22 enzymes catalyzing 20 metabolic reactions of the triterpenoid saponin pathway were identified (Additional file 1: Table S10). The presence of more unigenes in the G-type plants could partially reflect the higher heterozygosity among the sequenced individuals; polymorphisms within the alleles could produce multiple potential unigenes during the assembly process. Particularly evident were *MDD* and *LUP2*, which were represented by seven CL278.Contigs and five CL2531.Contigs, respectively (Additional file 1: Table S10). The authenticity of these sequences requires further experimental analysis. The expression abundances of these saponin synthesis genes were compared between G- and P-type plants (Fig. 3a and Additional file 1: Table S10); the genes upstream of *SE* showed similar expression patterns between the two genotypes. Unexpectedly, expression of genes for the bottom first and third enzymes, the glucosyl transferase (UGT73C11) and an oxidosqualene cyclases (OCS), was significantly upregulated, and these mRNAs accumulated by more than 10-fold in the P-type compared with the G-type plant. The over expression of these genes were also comfirmed by Q-PCR analysis, though the change levels were not as high as RNA-Seq displayed (Fig. 3b). One of them, UGT73C11, is known to be functional in the P-type plant [23]. The dramatic induction of these two enzymes could result from the P-type plants suffering more serious damage under insect infestation and because the regulator of this pathway is still functioning. However, some of the enzymes upstream of UGT73C11, most likely the uncharacterized enzyme-P450s, are perhaps dysfunctional or have gained new functions, resulting in no anti-DBM saponin being produced in P-type plants.

### Gene divergence and SNP and SSR markers between susceptible and resistant *B. vulgaris*

**Fig. 4:**
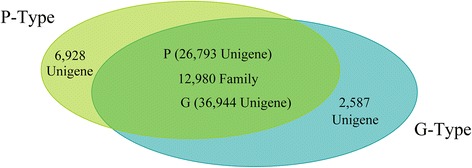
Venn diagram showing unique and homologous genes in P- and G-type *B. vulgaris*

The homologous genes were detected by BLAST searching the unigenes from P- and G-type *B. vulgaris* against each other with a cutoff E-value < e^−10^. The most similar unigenes within the P- or G-type were treated as paralogous genes, while the most similar ones between the P- and G-type were treated as orthologous genes. 12,980 gene families containing 26,793 P-type and 36,944 G-type unigenes were shared between P- and G-type *B. vulgaris* (Fig. 4). The 6,928 P-type and 2,587 G-type unique unigenes were mainly composed of 200–300-nt short sequences. The less clustered but more unique unigenes in P-type than in G-type reflected the fact that the P-type transcriptome contained a large fraction (7,857) of 200–300-nt short sequences. We then analyzed the SNP and SSR markers among the orthologous genes distinguishing the two plant genotypes. 38,397 SNPs were found within the 9,452 orthologous genes between the P- and G-type plants (the SNPs within each genotype are not included); thus, about 35.3 % unigenes contain SNPs, with an average of four SNPs per SNP-containing unigene (the detailed SNP list is shown in Additional file 1: Table S11). Among these SNPs, nucleotide transitions accounted nearly triple the number of transversions, and there were many more A/T transversions than G/C transversions (Table 3). The overall GC content is 47 % in this transcriptome. We detected 5,105 SSRs in the P-type transcriptome; the majority comprised mono-nucleotide-repeats (2,477) and triple-nucleotide-repeats (1,590), which represented 48.5 % and 31.1 % of the total SSRs (listed in Additional file 1: Table S12). The SSR-harboring unigene sequences were BLAST searched against the orthologous genes from the G-type plant. 1,657 SSRs with polymorphisms between the two genotypes were identified by a manual check. Among these, 98.4 % comprised mono- (793), double- (273) and triple-(564) nucleotide-repeats, which accounted for 47.9 %, 16.5 % and 34.0 % of the total, respectively (Table 4). From the polymorphic SSRs, 913 primer pairs were designed to detect SSRs with a length divergence of more than two nucleotides between the two genotypes (the primers and the product sequences are listed in Additional file 1: Table S13). As shown in Table 5, the SSRs with 3, 6, 9 and 12-bp variants comprised significantly larger fractions than the other types, suggesting that the SSR type and repeat variant that did not cause frameshift mutations were preferentially selected during evolution.

**Table 3:** Types of single nucleotide polymorphisms in Barbarea vulgaris

Mutant	Number	Percentage (%)
A/G	11,434	29.8
C/T	11,705	30.5
A/C	3,852	10.0
A/T	4,606	12.0
G/C	3,013	7.8
G/T	3,787	9.9
total	38,397	100

**Table 4:** Statistics of simple sequence repeats (SSRs) with polymorphisms in the P- and G-type Barbarea vulgaris

SSR type	Number	Percentage (%)
mono-nucleotide-repeats	793	47.86
double-nucleotide-repeats	273	16.48
triple-nucleotide-repeats	564	34.04
tetra-nucleotide-repeats	9	0.54
penta-nucleotide-repeats	10	0.60
hexa-nucleotide-repeats	8	0.48
Total	1,657	100

**Table 5:** Summary of simple sequence repeat (SSR) primers for detecting the polymorphisms between the P-and G-type Barbarea vulgaris

Variant (bp)	Number	Percent
2	183	20.04
3	346	37.90
4	106	11.61
5	26	2.85
6	138	15.12
7	10	1.10
8	11	1.20
9	42	4.60
10	10	1.10
11	2	0.22
12	19	2.08
>12	20	2.19
total	913	100

### Genetic diversity of *Barbarea* germplasm

**Fig. 5:**
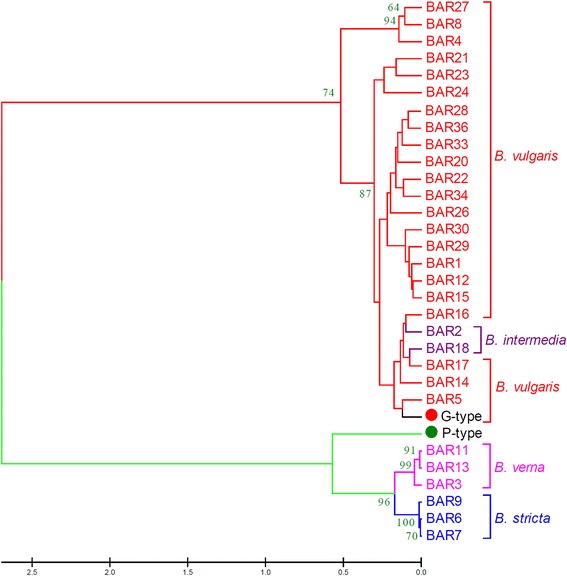
Bootstrapped unweighted pair-group method with arithmetic means (UPGMA) tree of *Barbarea* species. The P-type and G-type used for transcriptomics were compared to 30 seed bank accessions from four *Barbarea* species. The numbers on the branches indicate bootstraps

To test the utility of the SSRs produced in this study, 30 randomly chosen SSRs were used to investigate the genetic diversity of germplasms from the *Barbarea* genus*.* Thirty accessions assigned by the supplying seedbank to four species (*B. intermedia*, *B. stricta*, *B. verna*, and *B. vulgaris*) and derived from seven countries (Austria, Belgium, Germany, Ireland, Norway, Poland and Spain) were analyzed in addition to the two G- and P-type accessions used for transcriptomics (Additional file 1: Table S14). On PCR, 99.7 % of the primer pairs produced clear peaks on electrophoresis and generated 957 data points. The three (0.31 %) low-quality reactions were treated as missing values in the analyses. The 30 SSR markers generated 92 alleles in the population. Among these, 88 alleles displayed a frequency of more than 5 % in the total sample. Rare alleles could have been missed because of the small sample size. The summary statistics of the SSRs are shown in Table 6. The unweighted pair-group method with arithmetic means (UPGMA) tree was generated using the SSR data. Based on the tree topology, the germplasms could be clearly divided into 4 groups and the P-type alone (Fig. 5). The G-type and P-type accessions from this and a previous [29] transcriptomics study were widely separated as a logical consequence of their polymorphism for all markers used. The *B. verna* and *B. stricta* accessions were also separated onto different branches of the tree. Interestingly, the P-type from transcriptomics did not group with any other tested accession, while the G-type from transcriptomics grouped with all the remaining seed bank accessions of *B. vulgaris*, including twenty-two Western and Central European *B. vulgaris* accessions. All seed bank *B. vulgaris* accessions were also found to be resistant to the DBM. Some substructure was evident in the *B. vulgaris* group, including a distinct group of three accessions (4, 8, 27). However, the seed-bank assigned *B. intermedia* accessions were completely embedded in the G-type-like *B. vulgaris* germplasms. Morphological inspection of the two seed bank assigned *B. intermedia* accessions (as both rosette plants and flowering plants) revealed only modest morphological difference from *B. vulgaris* (shape of the top few leaves on the scapes) and both *B. intermedia* accessions showed full DBM resistance (as for the G-type).

**Table 6:** Characteristics of the 30 analyzed simple sequence repeat (SSR) markers and the diversity detected in 32 Barbarea accessions

Marker	Motif	Primer sequence (5′to 3′)	MAF	GNo	ANo	GD	H	PIC
**BV13-17**	(TC)6	ACAGGAAGAATGAGAAGAGCT	0.76	3.00	2.00	0.37	0.06	0.30
		TCTGTTTGTGGTTTTAATGGGC						
**BV13-18**	(T)12	TCAATGAAGACTATTTGAGATGCT	0.97	2.00	2.00	0.06	0.00	0.06
		CTTCAATAACTCCCTTACCAATGT						
**BV13-65**	(TGT)4	TCCTGCTGCTACTGTTGTTG	0.79	2.00	2.00	0.33	0.00	0.28
		TCAGAAACGGCATCCCTCTA						
**BV13-92**	(T)13	TGCGAATCAATCTTTCATTTGT	0.55	3.00	2.00	0.50	0.30	0.37
		TGTAGGTTTCTTTGGTTGCCA						
**BV13-98**	(TTC)5	GGCTTCTTCTCCTTTTACTTCCT	0.42	6.00	4.00	0.69	0.45	0.64
		GTGGACATGGTGGAAGGTTT						
**BV13-103**	(TGA)5	AAGCCCTCCAAACCAAGATG	0.85	5.00	3.00	0.27	0.06	0.25
		AAGCACAGATGATGCCAGAG						
**BV13-108**	(TCC)5	GGCGGTGGTGGATCTTATTA	0.88	2.00	2.00	0.21	0.00	0.19
		ATTTAGCACCACCACCTGAG						
**BV13-112**	(TTG)4	TGGTTTCGAGATGGGTTTCT	0.61	4.00	3.00	0.54	0.25	0.48
		ACCAATCCTCCAAGACAACA						
**BV13-123**	(T)8	GGCTGTGCAATCCTGAGTAA	0.88	6.00	3.00	0.22	0.09	0.21
		AGGAAGATGGATAATTTAAGCAGA						
**BV13-125**	(T)9	TCCACGAATCTTGCTTCTTTCT	0.86	3.00	2.00	0.24	0.15	0.21
		GACTATTTCTCTGTTGTTTTGAAGT						
**BV13-146**	(GAA)5	GGAGTCCTTCTGTCACTTCC	0.58	3.00	2.00	0.49	0.24	0.37
		CGTCATCGTCGGTGATACTC						
**BV13-174**	(A)11	CGTTCAAGGCACATCAGAGT	0.44	8.00	6.00	0.71	0.33	0.66
		TTCAAACACGGTAGTAGCGC						
**BV13-339**	(GGC)6	GTTCTTCTGCCGACGGTAAG	0.79	3.00	3.00	0.36	0.00	0.33
		GAGGAGGAGTTGAAGGCATG						
**BV13-370**	(GAA)5	GCAATGTGAGGATCATCACG	0.89	3.00	2.00	0.19	0.15	0.17
		AGTTGATGAAATCGCTGCAG						
**BV13-372**	(TGA)6	TGGCTTGGTTTGGTAGATGA	0.44	4.00	5.00	0.69	0.59	0.64
		GGTTTCTGCATCGACGAATG						
**BV13-375**	(GCG)6	CGTTCAAGGCACATCAGAGT	0.47	7.00	5.00	0.68	0.33	0.63
		TTCAAACACGGTAGTAGCGC						
**BV13-417**	(G)10	AGCCCTCTTGAGAACATTAAGA	0.70	3.00	2.00	0.42	0.06	0.33
		AAGTCTTTGTCTTTTGCTGC						
**BV13-425**	(TC)7	TAGAGGACGACGGAGACAAC	0.71	6.00	3.00	0.45	0.33	0.41
		TCATCATCAAAGGAGAATCGGA						
**BV13-426**	(CT)8	GAAACCTACACAAATAACAGAATGT	0.52	7.00	4.00	0.65	0.33	0.60
		GAACCGCATGTCTTAATTCGT						
**BV13-436**	(A)11	ACGATCATTTTGAGGTTTGAGA	0.38	8.00	4.00	0.71	0.36	0.66
		CTCTCTGGAATCACTGCACA						
**BV13-439**	(A)10	CCAACACCGAACGCATAAGA	0.68	3.00	2.00	0.43	0.09	0.34
		TCGCACTAATGTCTCCAACT						
**BV13-443**	(CTC)5	CGTTCCTTTACCCACTCGTC	0.39	4.00	4.00	0.71	0.33	0.66
		CGGTTTTAACAAGCTCGTCG						
**BV13-445**	(TCTT)5	CACATAACTCAGAACCGGACA	0.45	4.00	4.00	0.67	0.27	0.62
		TCCTCTGTTTCTCACTAAGTAGA						
**BV13-462**	(T)11	CTGCACAGACGACTCTTTT	0.83	3.00	2.00	0.28	0.03	0.24
		CCGATCCTCTTCTCCACCTT						
**BV13-485**	(T)12	TCGGTTTTGTTGCTTCCCAT	0.79	2.00	2.00	0.33	0.00	0.28
		TGCATTAGAGAGATTTCAAGTCC						
**BV13-509**	(T)10	ACGAAGGAGAAAGAACTTGCA	0.79	5.00	3.00	0.35	0.21	0.32
		TCTTTAGAGAGCAAGAAAGAGATAA						
**BV13-542**	(CGA)6	CAGGTTTCGCTCAGAGGAAG	0.33	7.00	5.00	0.74	0.47	0.69
		CTCTCGCGCTCTGAATCAAT						
**BV13-544**	(TCC)5	ACGCCAGGATGAATCTCAAC	0.77	3.00	2.00	0.35	0.09	0.29
		TTTCAGATTCGTCGCGGAAA						
**BV13-557**	(CCA)5	TAGCTTCCTCATTCCCACCA	0.88	2.00	2.00	0.21	0.00	0.19
		CCGTAATGAAACCTGGAGCA						
**BV13-558**	(GA)8	AGAGAGAAAACGAGAGAGAGAGA	0.81	3.00	2.00	0.30	0.25	0.26
		AAACATGGAACCAGCTTGCT						
**BV13-564**	(GGT)6	GAGGGAACGTTGGTGGTT	0.50	5.00	3.00	0.56	0.27	0.47
		ACGACGGCTGTTTACACTTT						

*MAF*, major allele frequency; *GNo*, genotype number; *ANo*, allele number; *GD*, gene diversity; *H*, heterozygosity; *PIC*, polymorphism information content.

## Discussion

The P- and G-types of *B. vulgaris* have been used extensively to explore plant-insect co-evolution, plant secondary metabolism, and for anti-insect gene identification [4–21, 25, 26, 29]. However, publicly released transcriptome data and molecular markers are extremely rare compared with other model plants. Following our previous transcriptome analysis of G-type plants, the present study presented the first P-type transcriptome data, which will be useful for further studies of the two genotypes. There were 14.7 % fewer assembled unigenes in the P-type compared with the G-type. One possible explanation is that the G-type sequencing produced nearly twice as many clean nucleotides as the P-type (Table 1), allowing the detection of more poorly expressed transcripts. However, the homology analysis of the unigenes revealed that the G-type contains significantly more paralogous genes than the P-type in each family (2.85:2.06) (Fig. 4). The targeted analysis of the triterpenoid saponin pathway also revealed more unigenes in the G-type than in the P-type (71:44) (Additional file 1: Table S10). Research in other plants has indicated that the genes involved in the triterpenoid pathways are often in gene clusters and can evolve new functions by duplication and reorganization [31–34]. Thus, it would be interesting to know whether the G-type plants have evolved a relatively more complicated genome with whole or partial genome duplication, and whether the genes of the triterpenoid saponin pathway are in a cluster in *Barbarea*. However, more data, such as genome sequencing, are needed to answer these questions.

The functional classification of the unigenes showed similar patterns between the P- and G-type transcriptomes, except in terms of the absolute number of each term, which was usually lower in the P-type compared with the G-type (Fig. 1), implying no bias derived from the transcriptome sequencing and assembly. There were significantly fewer DBM-affected genes in P-type plants, indicating that they have a less complex defense system against the DBM than the G-type. The major groups represented in the DBM-affected genes are those engaged in photosynthesis, nutrient processes, amino acid, lipid and carbohydrate transport and metabolism. The relationship between photosynthesis and insect herbivory response in plants was reviewed by ref. [35]. It is not easy to distinguish which of these effecs are direct responses to insect stimuli and which are indirect, reflecting the severe damage to the plant. However, the triterpenoid saponin pathway, which produces the major insect resistance compounds, was apparently efficiently induced by the DBM in P-type plants, indicating the insect sensing and induction networks of this pathway to be functional. It is thought that the anti-DBM triterpenoid saponins evolved in a common ancestor of *B. vulgaris* and *B. verna*, because these species share saponin based DBM (and flea beetle) resistance and the major saponin 3-*O*-cellobiosyl-hederagenin as the only known plants from the crucifer family [6, 15, 20]. It was also suggested that the P-type might have lost DBM resistance secondarily if truly included in the species *B. vulgaris*, but that the phylogeny of the two types needed to be tested by modern methods [6]. Our analyses of the putative saponin biosynthesis genes in the P-type suggest that the P-type plants possess the ability to produce other triterpenoid saponins than those conferring resistance to the DBM, because most of the genes of the pathway are expressed and show a positive response to insect stimuli. One possible explanation of the lack of DBM resistance is that in P-type plants certain enzymes in the biosynthesis pathway, such as P450s, are missing or not expressed, or P450s or OSCs have altered product profile, resulting in non-production of the specific anti-DBM saponins. Indeed, presence of unidentified saponin-like metabolites in the P-type has been suggested based on LC-MS analyses [17]. The putative P-type saponins and the present finding of differentially expressed saponin biosynthesis genes support that the P-type represents an advanced state derived from a G-type-like ancestor. It would be interesting to combine our expression-data with quantitative analysis of saponins in the infested P-type plants, but so far no-one has ever identified any of the putative P-type saponins, and the putative metabolites may require elaborate analytical methods [36].

Great efforts have been made in the map-based cloning of the insect-resistance genes in *B. vulgaris*. A genetic map has been constructed using 100 amplified fragment length polymorphisms (AFLP) and 31 SSR markers to map saponin genes and several other quantitative trait loci [26]. The authors identified 38,350 SSRs from the G-type transcriptome by 454 pyrosequencing. Among these, only 24 SSRs were finally used for map generation. One possible reason is that screening for polymorphic SSRs is inefficient using a single reference transcriptome. In the present study, two transcriptomes were compared and manually screened for polymorphic SSRs before primer design. More than 70 % of the 913 primer pairs produced clear polymorphic bands between the P- and G-type plants. Our method, although seemingly labor intensive, could reduce the costs of primer syntheses and the labor cost of experimental screening. Our high-quality ready-to-use SSRs and SNPs will be useful to construct high-density maps and for fine mapping of the saponin genes and other interesting trait loci.

In our genetic diversity analysis, because the SSRs we used are those that primarily distinguished the G- and P-type plants, the distance between the two types was expected to be magnified, and indeed the types were widely separated in the resulting tree. However, the fine clustering of the accessions in accordance with traditional taxonomy (except two *B. intermedia* accessions) suggested that the additional topologies of the tree represent genetic difference with biological significance. Many studies have shown that the P- and G-type plants are divergent in their morphology, phenology, cytology, phytochemical compounds and anti-insect capability [2–6, 11]. Recent studies also revealed a strong genetic separation and hybridization barrier between the two plant genotypes using five SSR markers, AFLP markers and a series of pollination experiments [12, 17, 37]. Our diversity analysis demonstrates similarity of the previously investigated G-type [5, 6, 29] to a large sample of Central and Western European *B. vulgaris* accessions in contrast to remarkable distinctness of the P-type accession investigated here and previously [5, 6]. The P-type is apparently recognized at the level of “variety” in classical taxonomy (var. *pubescens* Busch) [6, 38]. Any future taxonomic revision is yet undecided. A recent investigation across a hybrid zone of the two types in Eastern Europe found signs of hybridization and otherwise distinct geographical zones of each type, but the question of e.g. variety, subspecies or even species level separation has not been answered [17, 37, 38].

Interestingly, although P-type like accessions were not discovered in the investigated germplasm, another genetic substructure with no correlation to geographic origin was evident within *B. vulgaris*, with three accessions (4, 8, 27) forming a distinct group. This diversity deserves further investigation, and could represent established taxa [37] or yet unknown genotypes [38]. The finding of two *B. intermedia* accessions completely embedded in *B. vulgaris* and fully resistant to DBM was a surprise and could be a chance effect, considering genetic distinctness of *B. intermedia* in previous molecular investigations [37], near lack of flea beetle resistance in the species [6], and the well established status of *B. intermedia* [39]. Natural hybrids between *B. vulgaris* and *B. intermedia* are known [39], and as the two studied accessions were surprisingly similar to *B. vulgaris*, a hybrid nature of the seed bank-assigned accessions is another possible explanation for this finding.

Future studies employing unbiased molecular markers and integrating more germplasms of the *Barbarea* genus onto the tree may explain at what point the DBM resistance arose in a common ancestor of *B. vulgaris* and *B. verna* and possibly was lost in the P-type line [6] during the evolution of the *Barbarea*. The present detailed molecular genetic investigation of lack of DBM resistance, as compared to full DBM resistance [29], will contribute to understanding the molecular events associated with evolutionary loss of insect resistance.

## Conclusion

In conclusion, the present study reports the first P-type *B. vulgaris* transcriptomes from insect-free plants and those under DBM infestation. At the transcriptome level, the P-type plants reacted differently to the G-type under DBM infestation. Most of the genes of the triterpenoid saponins pathway are present and insect inducible in P-type plants. Many high-quality SNPs and SSR markers were identified based on P- and G-type transcriptome comparison. A subset of ready-to-use SSR primers was designed for genetic analysis of P- and G-type populations. Using the SSRs, further genetic diversity of *Barbarea* germplasms was revealed. These findings provide a basis for further investigation of the molecular biology and molecular ecology of the on-going evolution of insect resistance in *Barbarea* plants*.*

## Methods

### Plant materials

The *B. vulgaris* lines B4 (P-type) and B44 (G-type) (accession numbers as in [5]), which were pyrosequenced in the present and previous [5, 29] studies, respectively, were introduced from the University of Copenhagen, Denmark. The other 30 accessions used in this study were from the Leibniz Institute of Plant Genetics and Crop Plant Research (IPK), Germany. The passport information of these germplasms is listed in Additional file 1: Table S14.

Plant growth conditions were similar to our previous study [29]. Seeds were surface-sterilized in 1 % NaClO and sown in plastic pots (10 cm wide × 10 cm tall) filled with a mixture of peat soil (peat:moss:perlite:vermiculite soil = 3:2:1:1). Each pot contained one plant. Seedlings were placed in an artificial climate room with the temperature set at 25 °C (light) and 20 °C (dark), and 60 % relative humidity. Sodium lamps were used to maintain a minimum photosynthetically available radiation (PAR) of 225 μmol · m^−2^ · s^−1^, with a photoperiod of 16 h:8 h (L:D). Plants were watered regularly and fertilized with half-strength Hoagland’s nutrient solution. Plants aged 6 to 7 weeks were used for the DBM infestation assays.

### Insect treatment

DBM (*P. xylostella*) larvae were maintained on cabbage leaves in a climate-controlled cage at 25 °C, 12 h:12 h photoperiod and 50 % to 60 % relative humidity. Four DBM larvae of second to third instars were inoculated onto each fully expended leaf of the seedlings and allowed to stay on the leaves until the time of harvest. The control group comprised similar plants and was maintained under the same conditions but without exposure to DBM larvae. Five plants were used as biological replicates. Leaves from DBM-exposed plants and controls were harvested 4 h after the onset of herbivory, and flash-frozen in liquid nitrogen for future use.

### RNA isolation and sequencing

The TRIZOL reagent (Invitrogen, CA, USA) was used to isolate total RNAs from the liquid-nitrogen-frozen samples, according to the manufacturer’s instructions. BIOMARKER (Beijing, China) performed the Illumina sequencing. Total RNA (10 μg) was subjected to poly-A selection, fragmentation, random priming, and first and second-strand cDNA synthesis using the Illumina Gene Expression Sample Prep kit (Illumina, CA, USA). The cDNA fragments were subjected to an end repair process and then ligated to adapters. The products were enriched by PCR, and then the 350-bp fragments (270-bp insertion and 80-bp adapters) were gel-purified after 6 % TBE polyacrylamide gel electrophoresis. After denaturation, the single-chain fragments were fixed onto the Solexa Sequencing Chip (Flowcell) and subsequently grown into single-molecule cluster sequencing templates via in situ amplification on an Illumina Cluster Station. Double-end pyrosequencing was performed on the Illumina Genome Analyzer platform. The read-length is 101-bp for each end.

### Assembly

Raw reads produced from the sequencing machines were first subjected to purification by removal of adaptors and low-quality reads. The clean reads of infested and control plants were separately subjected to transcriptome de novo assembly using the short-read assembling program (Trinity) [40]. The longest assembled sequences were termed contigs. The paired-end reads were then mapped back to the contigs. Sequences without Ns and that could not be extended at either end were defined as transcripts. The TGI Clustering Tool (TGICL) [41] was then used to assemble the transcripts into unigenes. The unigenes from the treated and the control sample were clustered again; the longest ones from the two data sets were adopted to form a single set of non-redundant unigenes.

### Functional annotations

The program *getorf* from EMBOSS [42] was used to predict the open reading frames (ORF) of the unigenes. The deduced longest ORF was adopted as the coding region sequence (CDS) of the unigene. The CDSs were translated into amino sequences using the standard codon table. The functions of the unigenes were annotated by Nr, Nt, SwissProt, KEGG, COG, and GO methods. All unigene sequences were searched against these protein databases using BLASTX (*e*-value < 0.00001). Protein function information was predicted from the annotations of the most similar proteins in those databases.

### Expression levels

Unigene expression levels were calculated using the reads per kilobase per million (RPKM) method [43], the formula for which is RPKM = (1,000,000 * C) / (N * L * 1000), where RPKM(A) is the expression of gene A, C is the number of reads that uniquely align to gene A, N is the total number of reads that uniquely align to all genes, and L is the number of bases in gene A. Statistical comparisons between the treated sample and control was performed using IDEG6 software [44]. The general Chi squared method was used and the false discovery rate (FDR) was applied to determine the threshold of Q-value. Unigenes were considered differentially expressed (DEG) when the RPKM between the treatment and the control displayed a more than two-fold change, with an FDR less than 10^−2^.

The DEGs were then mapped to GO terms and KEGG pathways and followed with an enrichment analysis useing hypergeometric test to find over-represented GO terms and KEGG pathways. The algorithm used is described as follows:

$$ P=1-{\displaystyle \sum_{i=0}^{m-1}\frac{\left(\begin{array}{c}\hfill M\hfill \\ {}\hfill i\hfill \end{array}\right)\left(\begin{array}{c}\hfill N-M\hfill \\ {}\hfill n-i\hfill \end{array}\right)}{\left(\begin{array}{c}\hfill N\hfill \\ {}\hfill n\hfill \end{array}\right)}} $$

Where *N* is the number of all genes with GO or KEGG annotation; *n* is the number of DEGs in *N*; *M* is the number of all genes that are annotated to certain GO terms or KEGG pathways; *m* is the number of DEGs in *M*. The calculated p-value goes through Bonferroni Correction [45], taking a corrected p-value ≤ 0.05 as threshold.

### Orthologous gene analysis

The unigenes from the P-type and G-type transcriptomes were BLAST searched against each other with a cutoff value e^−10^. The best homologs within each genotype were designated as paralogous genes. The best homologs between the two genotypes were designated as orthologous genes.

### SNP mining

SOAPsnp [46] was used to analyze the SNPs between the P- and G-type transcriptomes. SNPs with a sequencing depth more than 10, a score more than 30 and the adjacent SNPs harboring an interval no less than 5 bp were selected.

### SSR mining

The MIcroSAtellite identification tool MISA [47] was used to identify and localize SSRs in unigenes longer than 1 Kb. The SSR-containing sequences were extracted with a 300-bp (if less than 300 bp, extracted from the end) fragment upstream and downstream of the SSR region sequence to facilitate primer design. For identification of SSRs that could distinguish the P- and G-type plants, the SSR repeats between the orthologous genes were checked. SSRs with polymorphisms were extracted and submitted for primer design using Primer 3.0.

### DNA extraction and SSR analysis

Newly formed leaves from five individual plants per accession were bulk sampled. The cetyl trimethylammonium bromide method [48] was used to extract genomic DNA. Thirty SSR markers were used in this study and the primer pairs are listed in Table 6. PCR amplifications were performed in a 15-μl volume containing 20–50 ng template DNA, 0.4 pmol primers, 0.5 U Taq enzyme and 1× PCR reaction buffer. Reactions were performed with an initial denaturation step of 5 min at 95 °C; followed by 35 cycles of 95 °C for 30 s, 57 °C for 45 s and 72 °C for 30 s; and a final extension at 72 °C for 10 min. The PCR products were then separated on an 8 % polyacrylamide gel electrophoresis and visualized with silver staining.

### Genetic diversity assessment

PowerMarker version 3.25 [49] was used to calculate the allele number, allele frequency, heterozygosity, genotype number, gene diversity and polymorphism information content (PIC). Tree topologies were constructed based on the bootstrapped UPGMA method within the MEGA software [50].

### Q-PCR analysis

The plant samples and RNAs were prepared using the same method as that for RNA-Seq. First-strand cDNAs were synthesized from 800 ng total RNAs using *EasyScript* One-Step gDNA Removal and cDNA Synthesis SuperMix (TransGen, Beijing, China) and diluted 20-fold as templates. The primers of the selected genes were as follows: HMGS-F (5′- GGGCGTCTTGAAGTAGGAAG-3′) and HMGS-R (5′- CAGTTCCACCATAGCAAGCA-3′); OSC-F (5′- GTCGGACGTCAAACATGGGA-3′) and OSC-R (5′- GCCACAAGAGATCACTGCAA-3′); UGT-F (5′- CCTTGCACATCATTCCGTTGG-3′) and UGT-R (5′- TGGCCAAGTGAGCAATGGAA-3′). The *Tublin* (Tublin-F: 5′- GGAGATGTTTAGGCGTGTG-3′ and Tublin-R: 5′- GCGTCTTGGTATTGCTGGT-3′) were used as internal controls. Experiments were performed on a StepOne™ Real-Time PCR System (Applied Biosystems, UK) using *TransStar* Green qPCR SuperMix (TransGen). The reaction volume was 20 μL, including 10 μL of *TransStar* Green qPCR SuperMix, 0.4 μL of 10 mM primer, 1.0 μL of the cDNA sample, 0.4 μL of Passive Reference Dye and 7.8 μL of dH_2_O. The following thermal cycling profile was used: 95 °C 10 min; 40 cycles of 95 °C for 15 s, 58 °C for 15 s, 72 °C for 10 s; 95 °C for 15 s, 60 °C for 1 min, 95 °C for 15 s. Three independent biological and technical replicates were performed. Data were analyzed using StepOne™ Software Version 2.0 (Applied Biosystems). The fold change was estimated in terms of threshold cycles by the 2^−ΔΔCT^ method [51].

### Data access

RNAseq data are available at EMBL/NCBI/SRA (accession number SRR1582492 and SRR1583630).

## Additional files

Additional file 1: Table S1. The statistic of P-type *Barbarea* Unigenes. **Table S2.** The Nt annotation of unigenes from P-type *B.vulgaris*. **Table S3.** The Nr annotation of unigenes from P-type *B.vulgaris*. **Table S4.** The Swissprot annotation of unigenes from P-type *B.vulgaris*. **Table S5.** The GO annotation of unigenes from P-type *B.vulgaris*. **Table S6.** The COG annotation of unigenes from P-type *B.vulgaris*. **Table S7.** The KEGG annotation of unigenes from P-type *B.vulgaris*. **Table S8.** The differentially expressed unigenes from P-type *B. vulgaris* with DBM infest and non-infest control. **Table S9.** The KEGG pathways affected by DBM in P-type *B. vulgaris*. **Table S10.** The expression profiles of saponin pathways in P- and G-type *B. vulgaris*. **Table S11.** The SNPs between P- and G-type *B. vulgaris*. **Table S12.** The SSR list from P-type *B. vulgaris*. **Table S13.** The SSR primer list for *B. vulgaris*. **Table S14.** The information list of *Barbarea* germplasm analyzed. Additional file 2: Figure S1. Expression (left) and fold-change (right) plots of transcripts of P-type *B. vulgaris*. **Figure S2.** Expression of photosynthetic related pathways response to diamondback moth. **Figure S3.** Expression of phenylpropanoid biosynthesis pathway response to diamondback moth. **Figure S4.** Expression of flavonoid biosynthesis pathway response to diamondback moth.

## Abbreviations

AACT: Acetyl-CoA acyltransferase
CDS: Coding region sequence
CMK: 4-diphosphocytidyl-2-*C*-methyl-D-erythritol kinase
COG: Clusters of orthologous groups
DBM: Diamondback moth
DEG: Differentially expressed gene
DMAPP: Dimethylallyl diphosphate
DXR: Deoxy-D-xylulose 5-phosphate reductase
DXS: Deoxy-D-xylulose 5-phosphate synthase
FDR: False discovery rate
FPP: Farnesyl diphosphate
FPS: Farnesyl pyrophosphate synthase
G3P: Glyceraldehyde 3-phosphate
GO: Gene ontology
GPP: Geranyl diphosphate
GPS: Geranylgeranyl pyrophosphate synthase
G-type: Glabrous type
HDR: HMB-PP reductase
IDI: IPI isomerase
HDS: (*E*)-4-Hydroxy-3-methyl-but-2-enyl pyrophosphate (HMB-PP) synthase
HMGR: 3-hydroxy-3-methylglutaryl CoA reductase
HMGS: 3-hydroxy-3-methylglutaryl CoA synthase
IPP: Isopentenyl diphosphate
KEGG: Kyoto encyclopedia of gene and genomes
MCT: 2-*C*-methyl-D-erythritol 4-phosphate cytidylyltransferase
MDD: mevalonic acid diphosphate decarboxylase
MDS: 2-*C*-methyl-D-erythritol 2,4-cyclodiphosphate synthase
MEP: 2-*C*-methyl-D-erythritol 4-phosphate
MK: Mevalonic acid kinase
MVA: Mevalonic acid
ORF: Open reading frames
2,3-OS: 2,3-oxidosqualene
OSC: Oxidosqualene cyclases
P450: Cytochrome P-450
PIC: Polymorphism information content
PMK: Phosphomevalonate kinase
P-type: Pubescent type
Q-PCR: Quantitative PCR
RPKM: Reads per kilobase per million
SE: Squalene epoxidase
SS: Squalene synthase
UGT: UDP-dependent glycosyl transferases
UPGMA: Unweighted pair-group method with arithmetic
