# Supplementary material for: Expression patterns, molecular markers and genetic diversity of insect-susceptible and resistant Barbarea genotypes by comparative transcriptome analysis
Source: BMC Genomics. 2015 Jul 1;16(1):486. doi: 10.1186/s12864-015-1609-y (PMC4487577; doi:10.1186/s12864-015-1609-y)
Supplement: Additional file 2: Figure S1. — Expression (left) and fold-change (right) plots of transcripts of P-type B. vulgaris. Figure S2. Expression of photosynthetic related pathways response to diamondback moth. Figure S3. Expression of phenylpropanoid biosynthesis pathway response to diamondback moth. Figure S4. Expression of flavonoid biosynthesis pathway response to diamondback moth. [file 12864_2015_1609_MOESM2_ESM.docx]

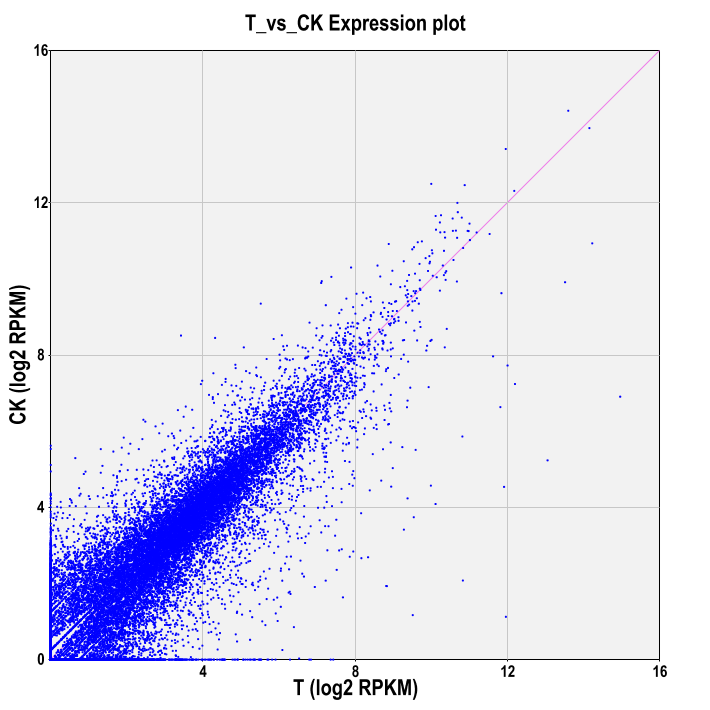

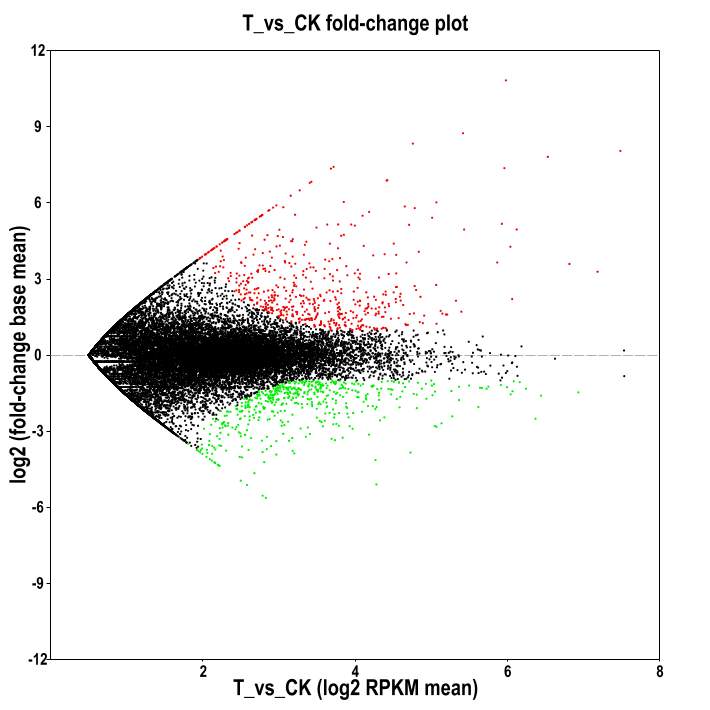


**Supplementary figure 1 Expression (left) and fold-change (right) plots of transcripts of P-type *B. vulgaris*.** T, DBM infect treated; CK, none infested control. The red plots indicate the up-regulated genes, the green indicate down-regulated.

**
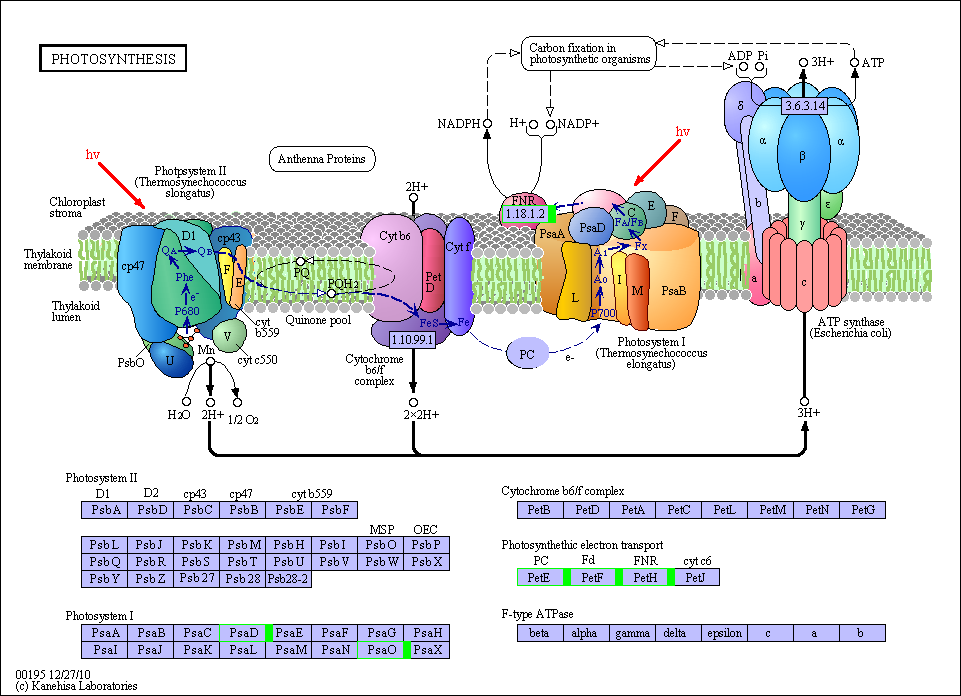
**

A


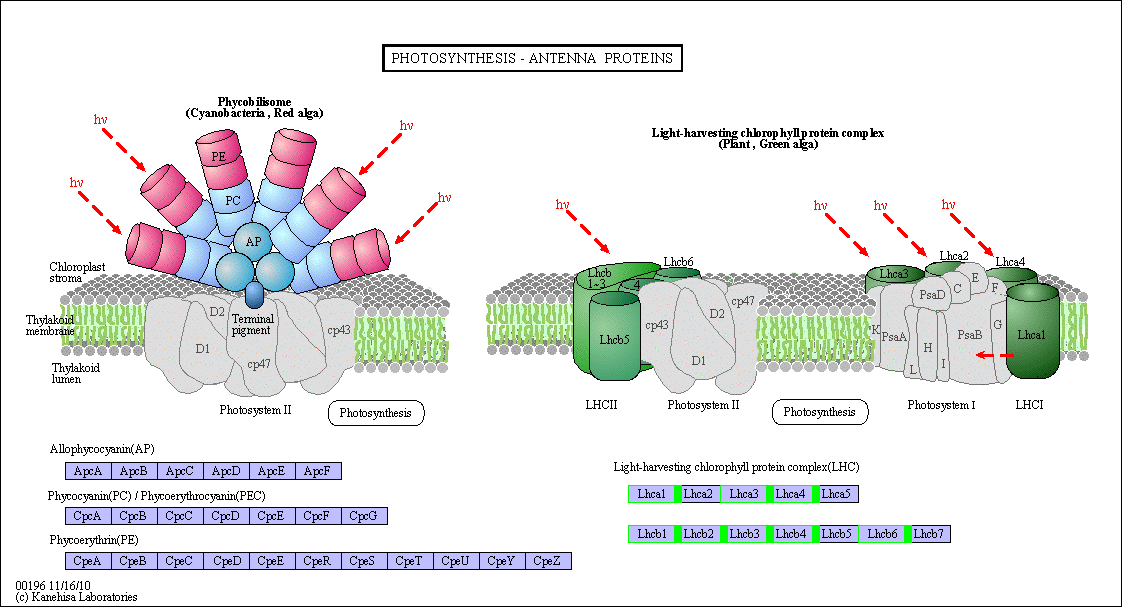


B


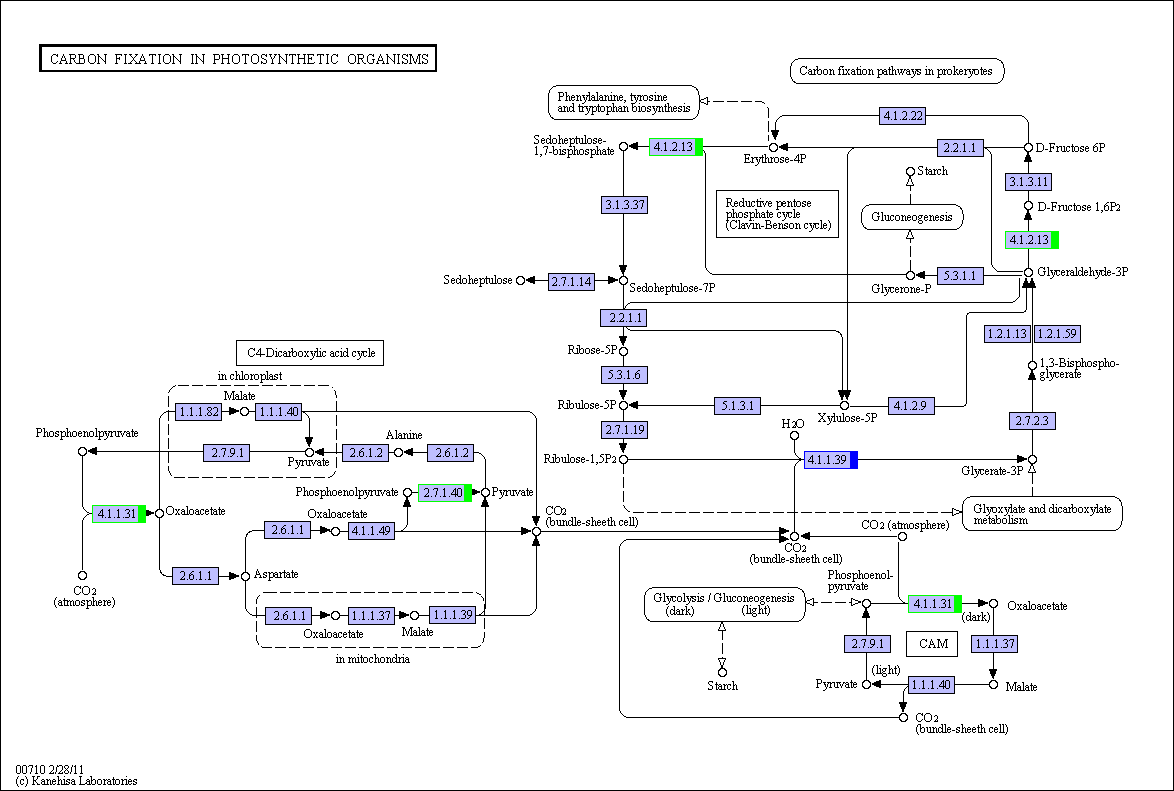


C

**Supplementary figure 2** **Expression of photosynthetic related pathways response to diamondback moth.** A, photosynthesis pathway (KEGG map00195: <http://www.genome.jp/dbget-bin/www_bget?map00195>). B, antenna proteins (KEGG map00196: <http://www.genome.jp/dbget-bin/www_bget?map001956>). C, carbon fixation in photosynthetic organisms (KEGG map00710: <http://www.genome.jp/dbget-bin/www_bget?map00710>) Green rectangles indicate the down- regulated genes.


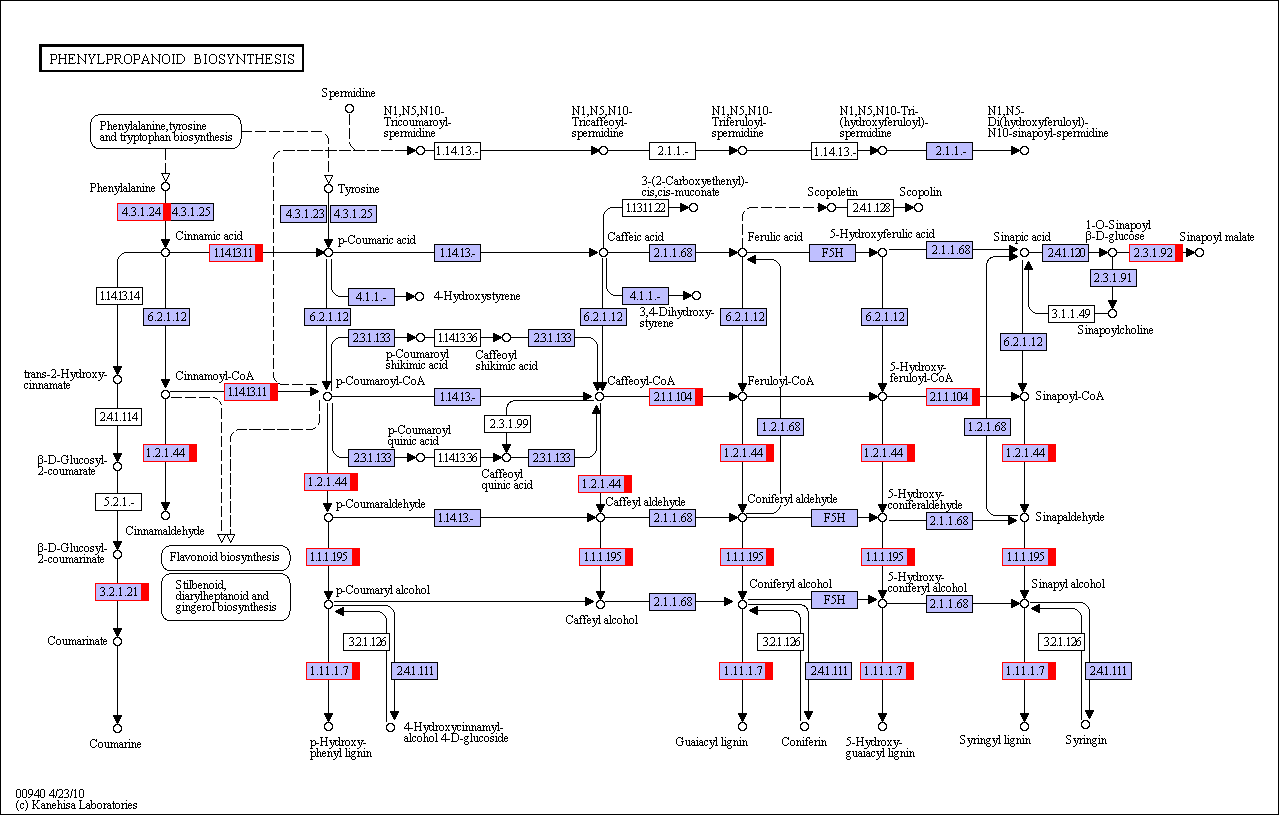


**Supplementary figure 3 Expression of phenylpropanoid biosynthesis pathway response to diamondback moth.** (KEGG map00940: <http://www.genome.jp/dbget-bin/www_bget?map00940>). Red rectangles indicate the up- regulated genes.


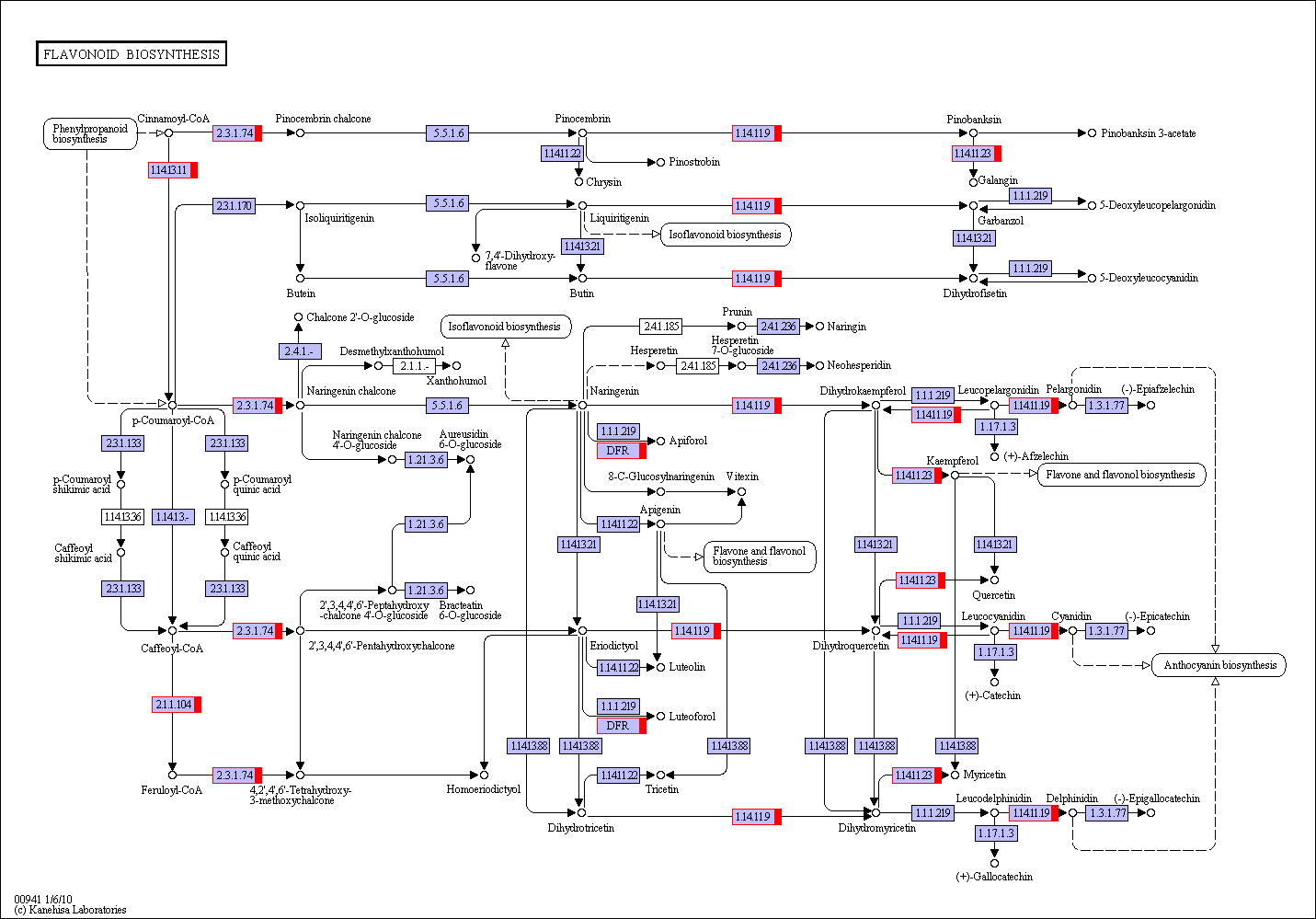


**Supplementary figure 4 Expression of flavonoid biosynthesis pathway response to diamondback moth.** (KEGG map00941: http://www.genome.jp/dbget-bin/www_bget?map009401). Red rectangles indicate the up- regulated genes.
